# Artificial intelligence for training and reporting infection prevention measures in critical wards

**DOI:** 10.3389/fpubh.2024.1442188

**Published:** 2024-10-16

**Authors:** Francesca Simioli, Anna Annunziata, Antonietta Coppola, Anna Iervolino, Mariacristina Boccia, Giuseppe Fiorentino

**Affiliations:** ^1^Respiratory Pathophysiology and Rehabilitation Unit, Monaldi Hospital, Naples, Italy; ^2^General Management, A.O. Dei Colli, Naples, Italy; ^3^Medical Management, A.O. Dei Colli, Naples, Italy

**Keywords:** hand wash, artificial intelligence, infection prevention and control (IPC), hospital acquired infection (HAI), critical care, Soapy, healthcare training

## Abstract

Healthcare associated infections (HAI) are a major concern. Infections and sepsis are prevalent causes of prolonged hospitalization, healthcare resources consumption, and mortality. Infection prevention and control (IPC) measures need to be encouraged with the purpose to become standard protocol. Hand hygiene represents the most important measure to prevent the spread of infections. Poor hand hygiene is often due to: the belief that the use of gloves replaces hand hygiene, lack of good example from colleagues and superiors, sinks that are difficult to access or insufficient in number, staff often too busy / not enough time, poor knowledge of guidelines and protocols. Artificial intelligence was recently integrated in hand washing devices; this can implement training on a daily basis but also is an objective method to assess the efficacy of the IPC measures adopted. The benefit–cost ratio of this approach needs to be discussed.

## Introduction

There is strong interest in the managing of Healthcare associated infections (HAI) and cleaning as a preventive method. Microorganisms such as vancomycin resistant enterococci (VRE), methicillin resistant *Staphylococcus aureus* (MRSA), multiresistant Gram-negative bacilli, norovirus and Clostridium spp. persist in hospital for days. These pathogens are often diffused by patients and staff, and also, they contaminate surfaces for days and increase the risk of acquisition for other patients. Environmental screening confirms repeated contamination of items, equipment, and general sites in bed spaces and rooms of colonized or infected patients and often throughout multiple clinical areas in a health care institution (1). Health care workers’ hands are liable to touch these contaminated surfaces during patient care, which increases the risk of onward transmission to others (2). Unrecognized environmental reservoirs may also act as a focus for outbreaks or ongoing sporadic transmission. Recent studies suggest that the risk of acquiring VRE, MRSA, *Acinetobacter* spp., *Pseudomonas* spp., or *C. difficile* is increased if a new admission is placed in a room previously occupied by a patient known to be colonized or infected with one of these pathogens (3–5). Environment and healthcare professionals play a key role in pathogen transmission. Survival percentage of each species or strains on floors and other surfaces could determine the rate of HAI risk for long stay patients.

A recent meta-analysis (6) showed that HAI sepsis is of major public health importance, and the burden is particularly high in intensive care units (ICU). There is an urgent need to improve the implementation of global and local infection prevention and management strategies to reduce its high burden among hospitalized patients. Sepsis can occur as a complication of infections acquired in the community, which is reported to represent up to 70% of all sepsis cases according to Reinhart et al. (7). It can also develop from HAIs that are mostly preventable by appropriate infection prevention and control (IPC) measures (8). According to a 2011 global report by the World Health Organization (WHO), HAIs prevalence varies between 5.7 and 19.1% hospital-wide (9). More recent data show that in Europe (10) and the USA (11) hospital-wide prevalence of HAIs is 6.5 and 3.2%, respectively. A multicenter prospective study in ICUs in Brazil suggested that HAIs relatively play a more significant role in epidemiological burden in low- and middle-income countries (12). Importantly, recent data showed that up to 55% of all HAIs can be prevented by the implementation of multifaceted IPC interventions (13), which would ultimately result in a significant reduction in hospital-acquired sepsis (HA sepsis) cases. However, most sepsis studies lack the differentiation between community-acquired and HA sepsis, and no systematic review on the global burden of HA sepsis has been conducted yet, including in the ICU setting.

Detergents and disinfectants can equally reduce spreading of pathogens but there is lack of quality evidence about cleanliness quality and its effect on clinical outcomes. Furthermore, cost-effectiveness is hard to evaluate.

## Aim

The aim of this paper is to discuss the potential role of artificial intelligence (AI) for training of healthcare professionals in critic departments of our hospital.

## Methods

Our hospital was equipped for a training period of 4 weeks with Soapy Clean Machine, an intelligent micro-station for hand washing. It is fully automatic, customizable and touch free, equipped with Artificial Intelligence, which allows to evaluate the performance of social and antiseptic hand washing of any user. Moreover, Soapy is recognized as part of the technologies to support the management of hospital infections. According to Italian law 6386/23 section III (President Travaglino), the burden to testify compliance to prevention measures by the healthcare personnel is due and at the expense of the General Management.

Three machines were tested in the departments of Neonatal Intensive Care, Pediatric Cardiology and Adult Respiratory Care. Soapy is an intelligent sink that allows you to monitor hand washing by checking whether users have correctly carried out the most effective preventive measure, according to the World Health Organization, against the spread of Covid-19 infection. At the end of the washing, the AI gives precise feedback, which is immediate on each step: if the user received the soap in his hands (REAGENT), if the Soapy has detected the 8 steps of hand washing that the operator has carried out in sequence (LATHERING – RUB & SCRUB), if the operator has carried out the rinsing properly (RINSING), percentage of accomplishment and effectiveness (SMILE WITH %). A smile indicates effective hand washing, and it is achieved if the process is at least 60% correct. The hardware of the Soapy Clean Machine includes two sonars, one on the top and one on the left side. It also includes a recording cam with a LED light and a thermometer that measures the body temperature before and after the washing process. The step-by-step procedure is shown to the user by an LCD screen. The dedicated software is innovative. It can see and recognize all passages, from emanation of the detergent to the middle of the hands, to the movements performed and the speed of rubbing. The system measures the surface that is actually rinsed.

The machine delivers a pleasant jet of instant hot water and uses a special patented hand cleaner, ensuring the correct duration of each wash cycle. Soapy is easy to install, uses biodegradable cartridges and ensures up to 95% savings in water and electricity and up to 60% in reagents. Among the numerous functions, the possibility of identifying users both individually and in groups through semi-biometric recognition and recording the time, place and number of washes – as well as measuring body temperature – to evaluate the accuracy of the procedures of hand cleaning. The device uses AI and the Internet connection to manage a touch-free guided cleaning system, including body temperature detection at each wash, which returns a report available in real time on the cloud platform “SoapyWisdom.”

It is important to specify that users did not receive a specific training with Soapy Clean Machine before this observation. This protocol itself is a test that aimed to eventually use AI for the purpose of divulging the WHO recommended 8 step hand washing protocol.

Insight on the Soapy technology and underlying algorithm are not the objective of the present discussion and are confidential.

## Results

**Figure 1:**
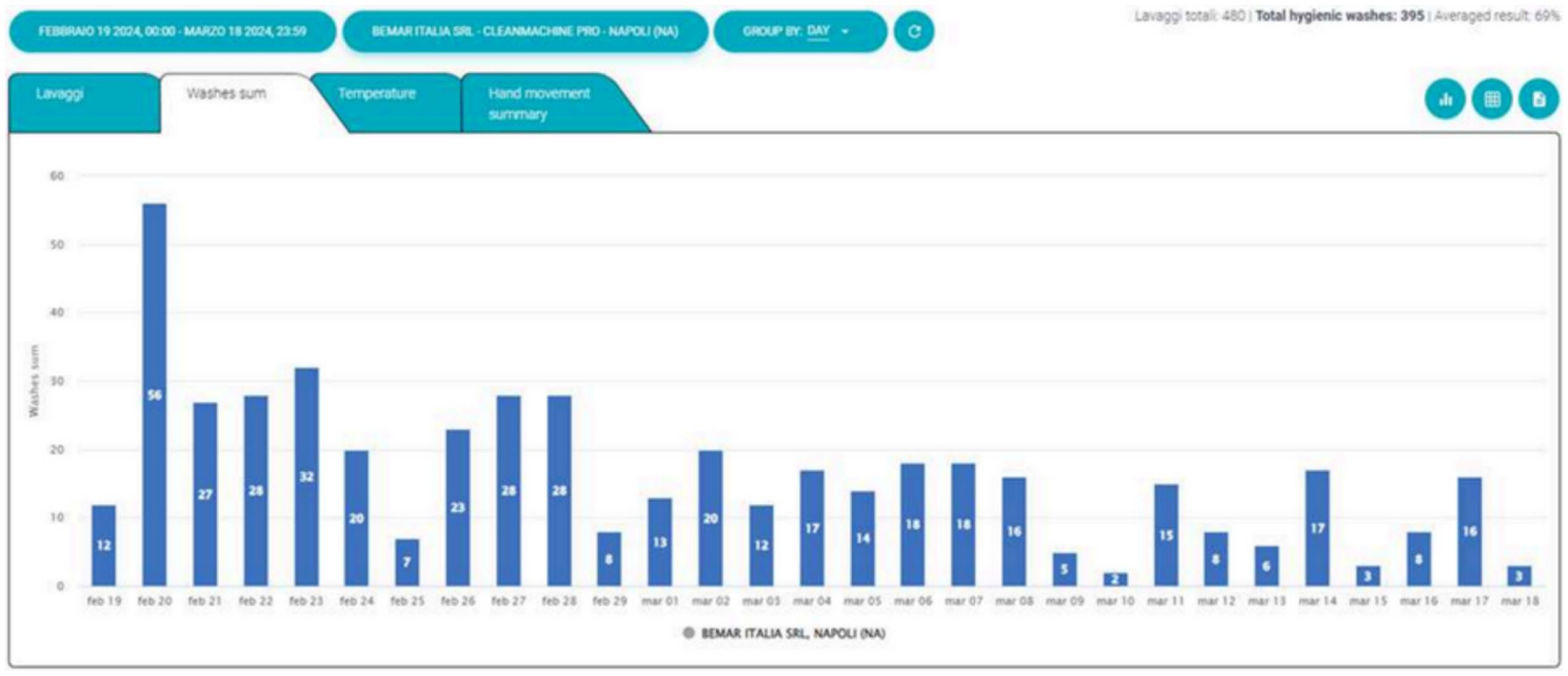
Total daily washings.

**Figure 2:**
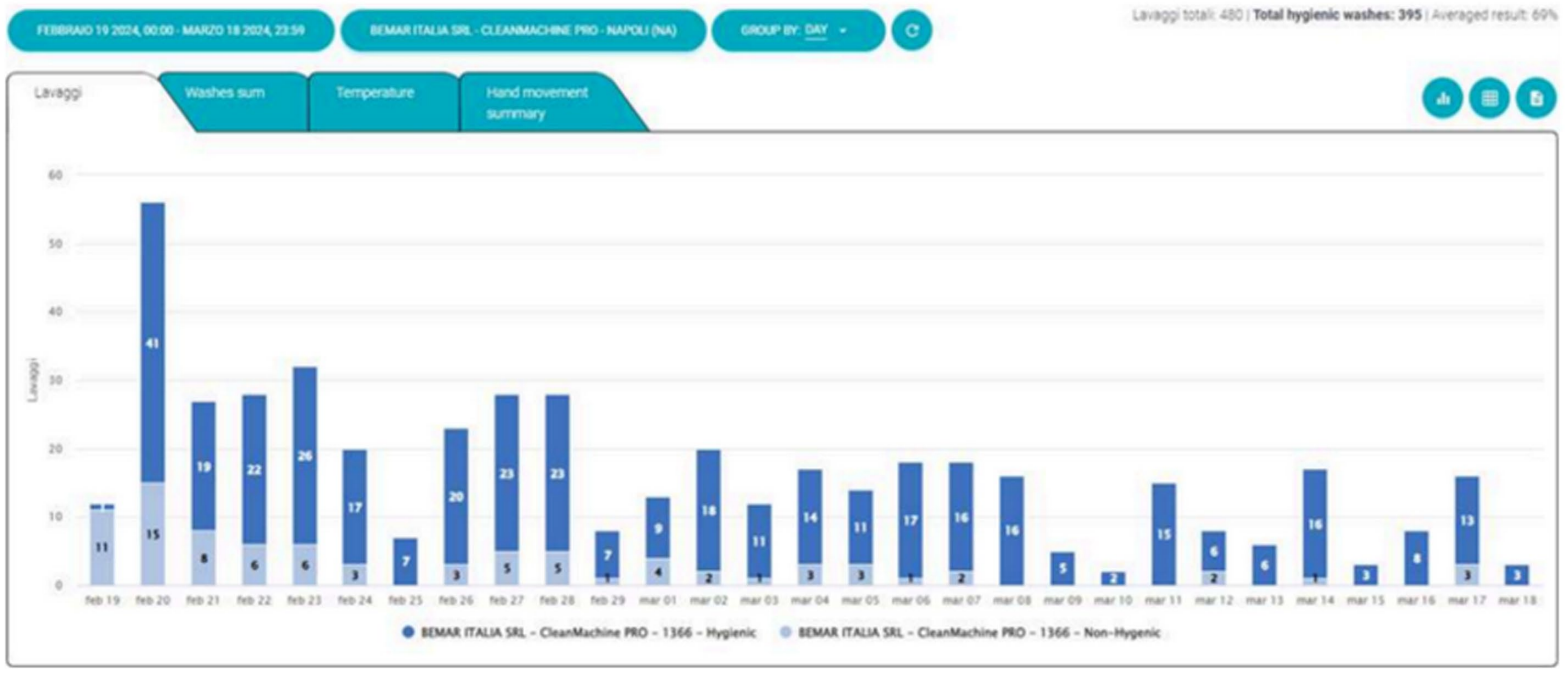
Total daily washings divided by hygienic (blue) and non-hygienic (light blue).

Three machines were tested in the departments of Neonatal Intensive Care, Pediatric Cardiology and Adult Respiratory Care. The Soapy machine recorded 1704 washings (Figure 1), of which 1,254 (74%) were evaluated as successful and hygienic. The mean overall performance value was 68% (Figure 2).

The errors were mostly recurring in some hand movements; whereas the AI suggests that it is possible that operators confuse the sequence of movements indicated on the display, and therefore do not carry out the right passage in the right way and at the right time. The picture (Figure 1) shows the most failed passages.

**Figure 3:**
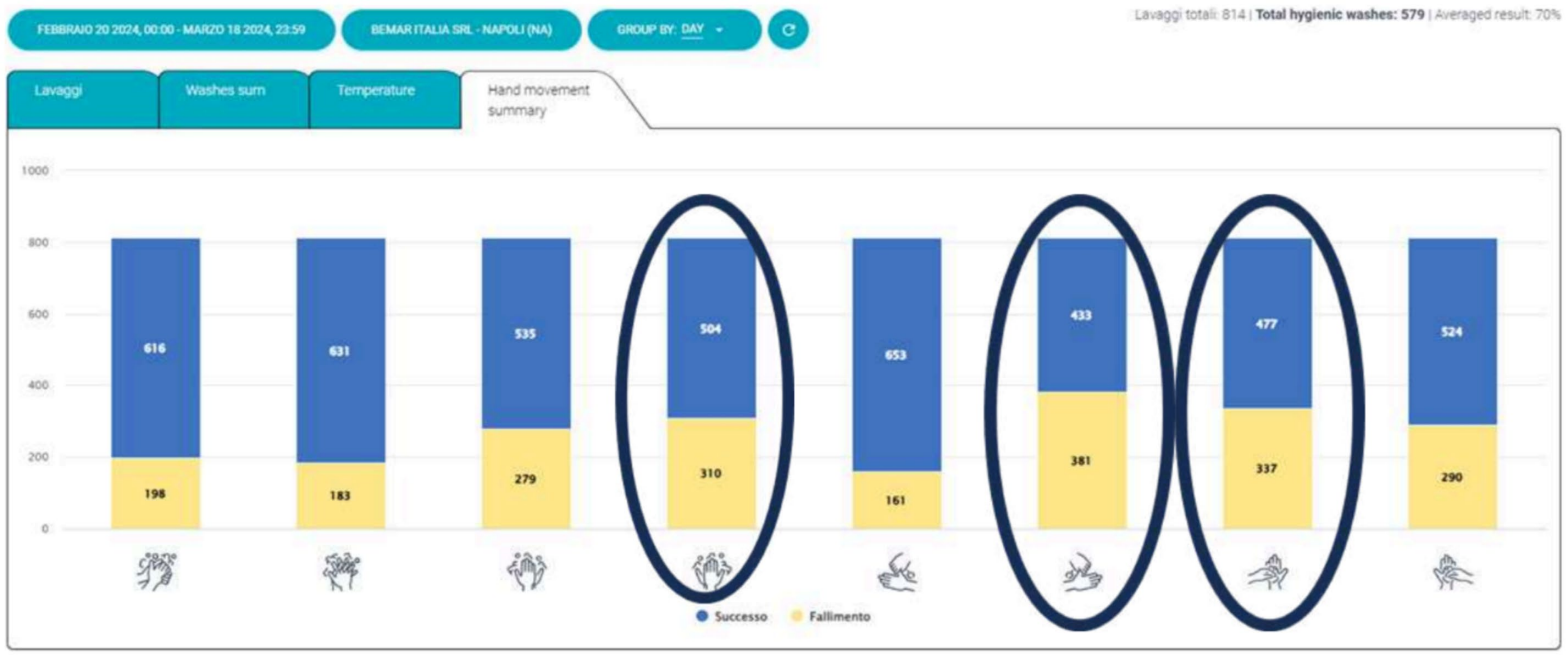
Step by step evaluation of hand movements during washing, divided by successful (blue) and failed (yellow).

According to the AI analysis, the entity of confusion by user’s increases with time, in fact most errors occurred in the latest passages of the sequence. But also, movements implying repetitive and specular gesture (left and right hand) innately cause a decline of attention and slowdown (Figure 3).

## Discussion

Nosocomial infections constitute a crucial moment for healthcare as, due to their action, sometimes completely silent, they manage to interfere with the health of patients as well as operators. They are the cause of prolonged hospital stays and sometimes result in death. A national prevalence study, conducted using the ECDC protocol, found a frequency of patients with an infection contracted during hospitalization equal to 6.3 per 100 patients present in hospital and 1 patient per 100 in home care. Not all HAIs are preventable, but prevention and control activities represent some essential interventions to reduce the impact of these infections and, more generally, to reduce the spread of antibiotic-resistant microorganisms; these activities can reduce the onset of HAI by a percentage greater than 50%.

According to the Center for Disease Control and Prevention (CDC) in Atlanta, hand hygiene represents the most important measure to prevent the spread of infections. Poor hand hygiene is often due to: the belief that the use of gloves replaces hand hygiene, lack of good example from colleagues and superiors, sinks that are difficult to access or insufficient in number, staff often too busy / not enough time, poor knowledge of guidelines and protocols.

Another aspect that should not be underestimated is the training of healthcare workers and the implementation of monitoring systems that allow management to have a complete picture of the current status of all hospital staff regarding hand hygiene and especially regarding their state of learning of this technique.

Based on our experience The Soapy Clean Machine system helps the operator to improve continuously as AI suggests the 8 steps to perform and gives an objective evaluation of the washing technique (execution of the 8 WHO steps indicated in the protocols). The AI allows healthcare workers, visitors and anyone with access to hospital departments, to watch in real time a step-by-step guide on how to accomplish a good washing. At the end of the hand washing, you can view your performance, as an overall and partial evaluation. It is our opinion that this helps both the initial learning and the continuous training process. Besides, the “SoapyWisdom” platform gives an objective record of the IPC adequateness in the hospital where the devices are used.

Nevertheless, some critical issues emerge from the data. In fact, the access to Soapy was found to be low compared to the number of operators per shift (on average there are 20 washes per day). This is probably because someone is still non confident with technology, but also because only one device was available for all the personnel. Moreover, the washing performance is poor in some steps of the 8 dictated by the WHO: some steps were not carried out correctly by the operators (see thumbs or palm/back). But this point remarks the importance of training, even in a periodic solution. Overall, the success percentage is greater than 50% in all departments. Potential solutions are to be researched in the technology of the device. Confidence with the machine is a major problem and a variable number of attempts is necessary to follow the procedure and be coordinated. This brings again to the importance of education. The decline of attention is another fundamental point. Additional signaling in between the steps could be introduced to regain attention. Vocal or visive feedback during the single steps can also help. We suggest any additional output that is else than monotony.

Remarkably these results are obtained after 4 weeks, which is a very short time. Long term use of AI device represents the only way to guarantee continuous learning. The control and feedback by AI give a daily refresh to healthcare professionals. Clinical practice is often chaotic and sometimes is makes very difficult to keep up with standards of IPC. Some procedures are not currently under objective evaluation in our country and are difficult to be proven. Nevertheless, IPC procedures massively impact the incidence of HAI. According to WHO, compliance with hand hygiene recommendations during health care delivery remains suboptimal around the world, with an average of 59.6% compliance levels in intensive care units up to 2018, and extreme differences between high income and low income countries (64.5% vs. 9.1%). Out of every 100 patients in acute-care hospitals, seven patients in high-income countries (HICs) and 15 patients in low- and middle-income countries (LMICs) will acquire at least one health care-associated infection during their hospital stay.

The use of this device with AI possibly highlights some critical issues which are currently not detectable and demonstrable in real life. The reporting software projected objective data on the current state of hand hygiene of users which, although high (greater than 50%), presents some problems. This made it possible to implement the training course on hand washing, focusing attention on the quality of washing and frequency, following WHO standards. No traditional method would have allowed us to have immediate and objective data, to allow a wide analysis of the scenario.

## Conclusion

Soapy Clean Machine elevates hand hygiene compliance by integrating its cutting-edge Hand Hygiene Monitoring System into a portable unit. This mobility allows healthcare providers to track and monitor hand hygiene compliance across various settings, ensuring consistent adherence to Leapfrog hand hygiene standards. The unit’s portability is particularly advantageous in dynamic environments where fixed systems are impractical, facilitating real-time hand hygiene monitoring wherever needed. The AI also offers comprehensive hand hygiene training and education, which are enhanced by gamification to engage users and improve learning outcomes. This mobile approach not only broadens the scope of hand hygiene compliance but also reinforces the importance of hand hygiene in preventing healthcare-associated infections.

## Data Availability

The original contributions presented in the study are included in the article/supplementary material, further inquiries can be directed to the corresponding author.

## References

[1] Lemmen SW Häfner H Zolldann D Stanzel S Lütticken R. Distribution of multi-resistant gram-negative versus gram-positive bacteria in the hospital inanimate environment. J Hosp Infect. (2004) 56:191–7. doi: 10.1016/j.jhin.2003.12.004. PMID: 15003666, PMID: 15003666

[2] Bhalla A Pultz NJ Gries DM Ray AJ Eckstein EC Aron DC. Acquisition of nosocomial pathogens on hands after contact with environmental surfaces near hospitalized patients. Infect Control Hosp Epidemiol. (2004) 25:164–7. doi: 10.1086/502369 14994944

[3] Huang SS Datta R Platt R. Risk of acquiring antibiotic-resistant bacteria from prior room occupants. Arch Intern Med. (2006) 166:1945–51. doi: 10.1001/archinte.166.18.1945 17030826

[4] Drees M Snydman DR Schmid CH Barefoot L Hansjosten K Vue PM. Prior environmental contamination increases the risk of acquisition of vancomycin-resistant enterococci. Clin Infect Dis. (2008) 46:678–85. doi: 10.1086/527394, PMID: 18230044

[5] Wilks M Wilson A Warwick S Price E Kennedy D Ely A. Control of an outbreak of multidrug-resistant Acinetobacter baumanii calcoaceticus colonization and infection in an intensive care unit (ICU) without closing the ICU or placing patients in isolation. Infect Control Hosp Epidemiol. (2006) 27:654–8. doi: 10.1086/507011 16807837

[6] Markwart R Saito H Harder T Tomczyk S Cassini A Fleischmann-Struzek C. Epidemiology and burden of sepsis acquired in hospitals and intensive care units: a systematic review and meta-analysis. Intensive Care Med. (2020) 46:1536–51. doi: 10.1007/s00134-020-06106-2 32591853 PMC7381455

[7] Reinhart K Daniels R Kissoon N Machado FR Schachter RD Finfer S. Recognizing sepsis as a global health priority—a WHO resolution. N Engl J Med. (2017) 377:414–7. doi: 10.1056/NEJMp1707170 28658587

[8] Saito H Kilpatrick C Pittet D. The 2018 World Health Organization SAVE LIVES: clean your hands campaign targets sepsis in health care. Intensive Care Med. (2018) 44:499–501. doi: 10.1007/s00134-018-5097-9, PMID: 29500700 PMC5924658

[9] World Health Organization. Report on the burden of endemic health care-associated infection worldwide. Geneva: World Health Organization (2011).

[10] Suetens C Latour K Karki T Ricchizzi E Kinross P Moro ML. Prevalence of healthcare-associated infections, estimated incidence and composite antimicrobial resistance index in acute care hospitals and long-term care facilities: results from two European point prevalence surveys, 2016 to 2017. Euro Surveill. (2018) 23:1800516. doi: 10.2807/1560-7917.es.2018.23.46.1800516 30458912 PMC6247459

[11] Magill SS O’Leary E Janelle SJ Thompson DL Dumyati G Nadle J. Changes in prevalence of health care-associated infections in U.S. hospitals. N Engl J Med. (2018) 379:1732–44. doi: 10.1056/nejmoa1801550, PMID: 30380384 PMC7978499

[12] Machado FR Cavalcanti AB Bozza FA Ferreira EM Angotti Carrara FS Sousa JL. The epidemiology of sepsis in Brazilian intensive care units (the Sepsis PREvalence assessment database, SPREAD): an observational study. Lancet Infect Dis. (2017) 17:1180–9. doi: 10.1016/s1473-3099(17)30322-5, PMID: 28826588

[13] Schreiber PW Sax H Wolfensberger A Clack L Kuster SP. The preventable proportion of healthcare-associated infections 2005–2016: systematic review and meta-analysis. Infect Control Hosp Epidemiol. (2018) 39:1277–95. doi: 10.1017/ice.2018.183, PMID: 30234463

